# Advanced Research on LysM Domain-Containing Proteins: Functional Mechanisms and Roles in Pathogenicity of Plant Fungi

**DOI:** 10.3390/ijms27062807

**Published:** 2026-03-20

**Authors:** Zhuoran Li, Xueming Zhu, Xiaoping Yu, Fucheng Lin

**Affiliations:** 1 College of Life Sciences, China Jiliang University, Hangzhou 310018, China; 2 State Key Laboratory for Quality and Safety of Agro-Products, Zhejiang Provincial Key Laboratory of Agricultural Microbiomics, Key Laboratory of Agricultural Microbiome (MARA), Institute of Plant Protection and Microbiology, Zhejiang Academy of Agricultural Sciences, Hangzhou 310021, China

**Keywords:** LysM effectors, phytopathogenic fungi, virulence factors, chitin-triggered immunity, immune evasion, disease control

## Abstract

Lysin motif (LysM) domain-containing proteins are widespread in prokaryotes and eukaryotes, and play crucial roles in microbe-host interactions. In recent decades, a large number of LysM domain-containing proteins have been identified and confirmed to participate in various biological processes, including microbial growth, fungal pathogenesis, and recognition of pathogens by plant immune receptors. Emerging evidence has shown that some LysM domain-containing proteins in plant pathogenic fungi have evolved as key virulence factors. They manipulate host immune responses mainly by interfering with the plant’s perception of chitin, a core pathogen-associated molecular pattern (PAMP) of fungal cell walls. However, the functions of LysM domain-containing proteins in plant pathogenic fungi have not been systematically summarized. In this review, we discuss the latest advances in the structural characteristics, classification, and functional mechanisms of these proteins, as well as their applications in plant disease control. We also propose the current challenges and future research directions in this field. This review aims to deepen the understanding of the molecular mechanisms underlying plant-fungal interactions mediated by LysM domain-containing proteins and provide theoretical references for developing novel and environmentally friendly strategies for fungal disease management in agriculture.

## 1. Introduction

Plant pathogenic fungi pose a severe threat to global food security and agricultural sustainability, causing devastating diseases that lead to substantial yield losses and quality deterioration of crops. For instance, *Magnaporthe oryzae* B.C. Couch, the causal agent of rice blast, infects rice plants worldwide and can result in 30–50% yield loss in epidemic years, threatening the food supply of billions of people [1]. Another notorious pathogen, *Zymoseptoria tritici* (Desm.) Quaedvl. & Crous, which causes Septoria tritici blotch (STB) in wheat, not only reduces grain yield but also produces pycnidiospores and ascospores that facilitate epidemic spread, thereby posing serious risks to global wheat production [2]. A recent global study on the impact of pathogens and pests on major food crops reported yield losses ranging from 17.2–30.0%, with rice suffering the highest losses (30.0%) [3]. These figures underscore the critical need to understand the molecular mechanisms underlying fungal pathogenicity and host plant immunity. In recent decades, extensive research has been conducted on these and other plant pathogenic fungi: studies on *M. oryzae* have clarified key infection processes such as appressorium formation and penetration [4,5], while research on *Z. tritici* has focused on the identification of effector proteins and the molecular basis of host-pathogen interaction and symptom development [6]. However, the coevolution between fungi and host plants has led to the emergence of more virulent fungal strains, making the management of fungal diseases an ongoing challenge.

To counteract invasion by plant pathogenic fungi and other pathogens, plants have evolved a sophisticated innate immune system, which is mainly composed of pattern-triggered immunity (PTI) and effector-triggered immunity (ETI) [7]. PTI is activated by the recognition of conserved pathogen-associated molecular patterns (PAMPs) by plant pattern recognition receptors (PRRs), serving as the first line of defense [8]. Chitin, a linear polymer of N-acetylglucosamine (GlcNAc) residues, is the major component of fungal cell walls and a typical PAMP that can be recognized by plant LysM domain-containing PRRs, such as the rice chitin-elicitor binding protein (CEBiP) and rice chitin-elicitor receptor kinase-1 (OsCERK1). These two proteins, encoded by *CEBiP* and *OsCERK1* genes respectively, cooperatively regulate chitin-triggered immunity (CTI) in rice [9]. CTI is characterized by a series of defense responses, including reactive oxygen species (ROS) burst, activation of mitogen-activated protein kinase (MAPK) cascades, and upregulation of defense-related gene expression [10].

However, phytopathogenic fungi have evolved sophisticated strategies to evade and suppress plant PTI, with the secretion of LysM domain-containing effectors being one of the most conserved, effective and well-characterized mechanisms [11]. The LysM domain was first identified in a bacteriophage lysozyme, and subsequent studies have shown that LysM domain-containing proteins in fungi can specifically bind to chitin and its derivatives [12,13]. Since the first LysM-containing effector Ecp6 was identified from *Cladosporium fulvum* Cooke in 2008 [14], numerous LysM domain-containing proteins have been characterized in various plant pathogenic fungi, such as *M. oryzae*, *Z. tritici*, *Colletotrichum higginsianum* Sacc., *Verticillium dahliae* Kleb., *Rhizoctonia solani* J.G. Kühn, and *Clonostachys rosea* (Link) Schroers [15,16,17,18,19,20]. These proteins exhibit diverse structures and functions, and their roles in fungal pathogenicity have been extensively studied.

In recent years, structural biology, genomics, and proteomics technologies have advanced rapidly, leading to significant progress in elucidating the molecular mechanisms by which LysM domain-containing proteins mediate fungal pathogenicity. The crystal structure of Ecp6 revealed a unique chitin-binding mechanism mediated by intramolecular LysM dimerization [21]. Comparative genomics studies have uncovered the evolutionary diversity of LysM domain-encoding genes among different fungal species [22]. Meanwhile, phosphoproteomics analyses have provided insights into the signal transduction pathways regulated by LysM domain-containing proteins [23]. Recent years have witnessed remarkable advances in this field, including the discovery of novel LysM domain-containing effectors with unique domain architectures, and the elucidation of host-specific recognition mechanisms at the atomic level [24]. In addition, the application of LysM domain-containing protein-related research in plant disease control has also achieved positive progress, such as the successful use of host-induced gene silencing (HIGS), a strategy in which transgenic plants produce small RNAs to specifically silence fungal genes, to silence fungal LysM domain-encoding genes and enhance plant resistance [25,26,27,28].

Over the past decade, the roles of LysM domain-containing proteins in microbial pathogenesis and symbiosis have been extensively reviewed [29]. These studies have established a broad framework for understanding LysM protein functions across kingdoms, particularly in the context of plant-microbe interactions [30]. However, existing reviews have primarily focused on evolutionary conservation or symbiotic signaling, with limited emphasis on the mechanistic diversity and virulence-specific roles of LysM effectors in plant pathogenic fungi. Recent advances in structural biology, comparative genomics, and effector biology have unveiled new layers of complexity, including intramolecular dimerization, host-specific recognition, and co-evolutionary dynamics between fungal effectors and plant receptors. Therefore, this review provides an updated synthesis focused exclusively on plant pathogenic fungi, systematically summarizing their structural characteristics, classification, functional mechanisms, representative species studies, application in disease control, and future perspectives, with an emphasis on the latest achievements from 2020–2025. This work aims to deepen the understanding of the molecular basis of fungal virulence and plant–fungal interactions, and to provide new ideas for the development of efficient and environmentally friendly fungal disease control strategies.

## 2. Structural Characteristics of LysM Domains

**Figure 1:**
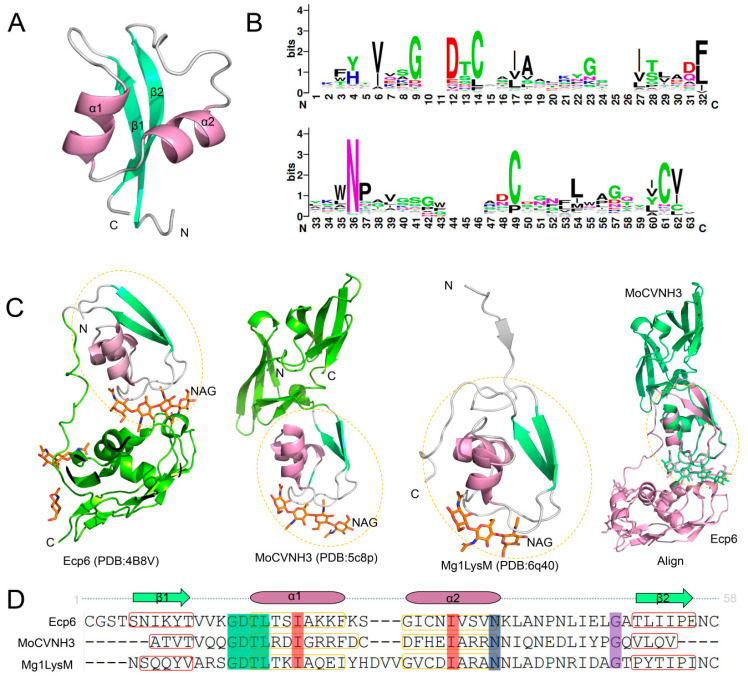
Structural characteristics of LysM domains in pathogenic fungi. (**A**) The structure of the LysM1 domain in Ecp6 from *C. fulvum*. The α-helices (α1, α2) and β-sheets (β1, β2) are labeled with pink and green colors, respectively. (**B**) WebLogo analysis of LysM domains in different fungal species. (**C**) The crystal structure models of Ecp6 (PDB:4B8V), MoCVNH3 (PDB:5c8p), Mg1LysM (PDB:6q40), and the alignment of MoCVNH3 and Ecp6. The α-helices, β-sheets, and N-acetylglucosamine (NAG) ligand-binding sites are labeled with different colors. (**D**) Sequence alignment of LysM domains in MoCVNH3, Ecp6 and Mg1LysM. Structural annotations are indicated above the sequences for MoCVNH3, Ecp6 and Mg1LysM, respectively.

The LysM domain is a conserved carbohydrate-binding module consisting of approximately 50 amino acid residues, with a typical βααβ secondary structure [31]. In this structure, two α-helices are stacked on the same side of an anti-parallel β-sheet, forming a hydrophobic pocket that specifically binds to GlcNAc-containing molecules, such as chitin, peptidoglycan, and chitosan. The amino acid sequence of the LysM domain is relatively conserved, especially the N-terminal and C-terminal regions, which are crucial for maintaining structural stability and ligand-binding activity (Figure 1B). For example, the first 16 residues and the last 10 residues of the LysM domain are highly conserved, and mutations in these regions often lead to the loss of chitin-binding ability. Unlike prokaryotic LysM domains, which rely on hydrogen bond networks to maintain their tertiary structure, eukaryotic LysM domains often contain conserved cysteine residues that form disulfide bonds, thereby enhancing structural stability. In addition, multiple LysM domains in the same protein are usually separated by flexible linkers rich in serine, threonine, and proline residues, which enable the LysM domains to adjust their spatial orientation for ligand binding.

Fungal LysM proteins exhibit significant structural diversity, mainly reflected in the number and arrangement of LysM domains. According to genomic analyses, fungal LysM proteins can contain 1 to 7 LysM domains, with most containing 1 to 3. In *C. fulvum* strains, Ecp6 contains three LysM domains (LysM1, LysM2, and LysM3), while Mg1LysM has only one LysM domain [32]. The structural diversity and ligand-binding mechanisms of LysM domains are depicted in Figure 1. The crystal structure of Ecp6 revealed a unique intramolecular dimerization mechanism of LysM domains [33] (Figure 1A). LysM1 and LysM3 of Ecp6 form a composite chitin-binding groove through intramolecular interaction, which binds to chitin oligosaccharides with ultra-high affinity (picomolar level), much higher than that of plant PRRs (nanomolar level) [34]. In contrast, the single LysM domain of Mg1LysM forms homodimers in both chitosan-dependent and chitosan-independent manners, and the chitosan-induced oligomerization of Mg1LysM enables it to form supramolecular structures, thereby protecting fungal cell walls from chitinase hydrolysis [35]. Most functionally characterized fungal LysM effectors contain two LysM domains, such as *M. oryzae* Slp1, *V. dahliae* Vd2LysM and *C. higginsianum* ChELP1/2. Structural studies have shown that these two-domain LysM effectors do not undergo intramolecular LysM dimerization like Ecp6, but form intermolecular dimers [36,37]. Sequence alignment of LysM domains from representative proteins, such as MoCVNH3, Ecp6, and Mg1LysM, reveals conserved structural features (Figure 1D). Although their chitin-binding affinity is lower than that of Ecp6, they can form polymer complexes in the presence of chitin, which may precipitate at the infection site to eliminate chitin oligosaccharides, thereby inhibiting CTI activation. Recent studies have further expanded the structural diversity of fungal LysM proteins; for instance, AlphaFold3-driven structural prediction, combined with experimental validation, has uncovered cryptic chitin-binding sites in several previously uncharacterized LysM proteins (Figure 1C), providing new insights into the structural basis of their functional versatility [38,39].

## 3. Classification and Functional Mechanisms of Fungal LysM Proteins

It is important to distinguish between LysM domain-containing proteins and LysM effectors. While all LysM effectors are LysM domain-containing proteins, not all LysM domain-containing proteins function as effectors. Some LysM proteins are involved in fungal development, cell wall integrity, or other cellular processes, and lack the typical features of secreted effectors, such as a signal peptide or virulence function [16]. Thus, throughout this review, “LysM effector” refers exclusively to secreted proteins that counteract host immunity, whereas “LysM domain-containing protein” is used in a broader sense to include the entire family, irrespective of functional characterization.

**Figure 2:**
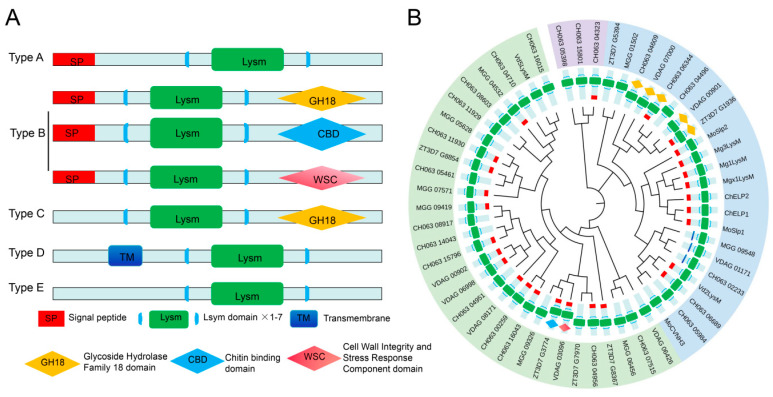
Classification of LysM-containing proteins in pathogenic fungi. (**A**) Five-category classification system (Type A-E) for LysM proteins in plant pathogenic fungi. The domain characteristics of each type of protein are indicated in the schematic diagram: Signal peptide (SP), LysM domain (1–7 copies), Transmembrane domain (TM), Glycoside Hydrolase Family 18 domain (GH18), Chitin Binding Domain (CBD), Cell Wall Integrity and Stress Response component domain (WSC). (**B**) Maximum likelihood phylogenetic tree of 108 LysM domains from 4 fully sequenced plant pathogenic fungal species: *Magnaporthe oryzae* 7015, *Zymoseptoria tritici* (strain ST99CH_3D7), *Colletotrichum higginsianum* (strain IMI 349063), and *Verticillium dahliae* (strain VdLs.17). Phylogenetic analysis was performed in MEGA 12 by aligning the sequences using ClustalW, with Maximum Likelihood, 1000 Bootstraps, and iTOL v7 was used to draw the phylogenetic tree. Typical representative proteins are labeled on the corresponding branches, reflecting the functional conservation and species-specificity of each type of protein.

The classification and phylogenetic relationships of fungal LysM proteins are summarized in Figure 2. Based on their domain composition and subcellular localization, fungal LysM proteins can be divided into five categories [24]: (1) Type A proteins (secreted LysM effectors): The largest category, containing only LysM domains with an N-terminal signal peptide and no transmembrane domains. (2) Type B proteins (multi-domain secreted proteins): Containing LysM domains and other functional domains, such as Glycoside Hydrolases family 18 domain, Chitin Binding domain and Cell Wall Integrity and Stress Response component domain, with a signal peptide and no transmembrane domains. (3) Type C proteins (intracellular multi-domain proteins): Containing LysM domains and other functional domains, but lacking signal peptides and transmembrane domains. (4) Type D proteins (membrane-associated proteins): Containing LysM domains and one or more transmembrane domains, including membrane proteins and transporters. (5) Type E proteins (minimal LysM proteins): Containing only LysM domains and lacking signal peptides, transmembrane domains, and any other identifiable functional motifs. The simplest architecture containing only a single LysM motif is conserved across all kingdoms [22], suggesting that similar minimal LysM modules may exist in fungi (Figure 2A). The representative LysM proteins corresponding to each type are listed in Table 1, illustrating their diverse domain architectures across different fungal species. The selected species include *Magnaporthe oryzae*, *Zymoseptoria tritici*, *Colletotrichum higginsianum*, *Verticillium dahliae*, *Fusarium verticillioides*, *Cladosporium fulvum*, *Colletotrichum gloeosporioides*, *Colletotrichum lindemuthianum*, *Colletotrichum fructicola*, and *Rhizoctonia solani.* These species were selected based on their economic importance, phylogenetic diversity (spanning Sordariomycetes, Dothideomycetes, and Agaricomycetes), the availability of high-quality genome assemblies, and prior functional characterization of LysM effectors [24]. This broad representation enables comparative analysis across distinct infection strategies and provides a foundation for understanding the functional evolution of LysM proteins in plant pathogenic fungi. Among these LysM proteins, Type A proteins (secreted LysM effectors) are the most extensively studied due to their direct role in suppressing host immunity, while Type B proteins (cell wall-associated LysM proteins) are also important for fungal pathogenicity, as they are involved in fungal cell wall synthesis and degradation. The functions of Type C–E proteins in pathogenic fungi have not been well studied, mainly due to their low expression levels and the lack of obvious phenotypes after gene knockout. Recent studies have proposed a revised classification system that incorporates the evolutionary origin of LysM domains [22,24]. This system divides fungal LysM proteins into orthologous groups based on phylogenetic analysis, which better reflects their functional conservation and divergence across different fungal lineages (Figure 2B).

The core functional mechanisms of fungal LysM proteins in pathogenicity mainly include chitin sequestration, inhibition of PRR activation, protection of fungal hyphae, and regulation of fungal development. Chitin sequestration is the primary virulence function of most secreted LysM effectors during fungal infection. During fungal infection, plant chitinases degrade the fungal cell wall, releasing chitin oligosaccharides [40]. Fungal LysM effectors can bind to these chitin oligosaccharides with high affinity, preventing them from being recognized by plant PRRs, thereby inhibiting the activation of CTI [41]. For example, Ecp6 from *C. fulvum* can efficiently sequester chitin fragments, and the deletion of Ecp6 significantly reduces the virulence of *C. fulvum* on tomato plants. Similarly, Slp1 from *M. oryzae* and Mg3LysM from *Z. tritici* can also sequester chitin oligosaccharides and suppress CTI, which are essential for fungal virulence [42]. Some LysM effectors not only bind to chitin but also directly interact with plant PRRs, interfering with their activation. In *C. fulvum*, Ecp6 can bind to the extracellular LysM domains of plant CEBiP and CERK1, preventing the formation of the CEBiP-CERK1 receptor complex, which is critical for chitin signal transduction [43]. This direct interference with PRR activation provides an additional layer of immune suppression for fungi. Recent studies have demonstrated that fungal LysM effectors can specifically interact with the extracellular domain of plant CERK1, inducing its ubiquitination and degradation, thereby blocking CTI signal transduction [44,45].

Many LysM proteins localize to the fungal cell wall, forming a protective layer that shields chitin from degradation by plant chitinases [46]. Mg1LysM from *Z. tritici* forms supramolecular structures on the fungal cell wall through oligomerization, which physically blocks the access of plant chitinases to chitin. Similarly, BdLM1 from *Botryosphaeria dothidea* (Moug.) Ces. & De Not. and LtScp1 from *Lasiodiplodia theobromae* (Pat.) Griffon & Maubl. can protect fungal hyphae from chitinase hydrolysis, thereby enhancing fungal virulence. In addition to immune suppression, some LysM proteins are involved in fungal development processes, such as conidiation, appressorium formation, and hyphal growth [47]. Cg2LysM from *Colletotrichum gloeosporioides* is involved in conidiation, appressorium formation, and melanin synthesis, and the deletion of *Cg2LysM* leads to reduced conidiation and appressorium penetration ability. Recently, a study of 18 putative LysM proteins in *Penicillium* spp. found that the expression levels of *PeLysM1*, *PeLysM2*, *PeLysM3*, and *PeLysM4* were significantly increased during pathogen infection, but their gene knockout did not affect fungal virulence. PeLysM3 was also proved to regulate spore germination and growth rate [48] (Table 1).

**Table 1:** LysM domain-containing proteins in representative plant pathogenic fungi. The table includes organism, protein name, domain architecture (number of LysM domains and additional domains), presence of signal peptide (SP) and transmembrane domain (TM), along with relevant references.

Organism	Name	Lysm Domains	SP	TM	References
*Magnaporthe oryzae* 7015	Slp1	2	Y	-	[19]
	MoCVNH3	1	-	-	[37]
	Slp2	2	Y	-	[19]
*Zymoseptoria tritici* (strain ST99CH_3D7)	Mg1LysM	1	Y	-	[20,35,49]
	Mg3LysM	3	Y	-	[20,49]
	Mgx1LysM	1	Y	-	[20,49]
*Colletotrichum higginsianum* (strain IMI 349063)	ChELP1	2	Y	-	[18,34]
	ChELP2	2	Y	-	[18,34]
*Verticillium dahliae* (strain VdLs.17/ATCC MYA-4575/FGSC 10137)	Vd2LysM	2	Y	-	[17,34]
	Vd5LysM	5		-	[16]
*Fusarium verticillioides* 7600	FvLcp1	3	Y	-	[50]
*Cladosporium fulvum*	Ecp6	3	Y	-	[51]
*Colletotrichum gloeosporioides*	Cg2LysM	2	Y	-	[47]
*Colletotrichum lindemuthianum* (strain race κ)	CIH1	2	Y	-	[18]
*Colletotrichum fructicola*(strain CGMCC3.17371)	CfLysM2	2	Y	-	[52,53]
*Rhizoctonia solani* AG2-2IIIB	RsLysM	2	Y	-	[15]

## 4. Application of LysM Protein Research in Plant Disease Control

**Figure 3:**
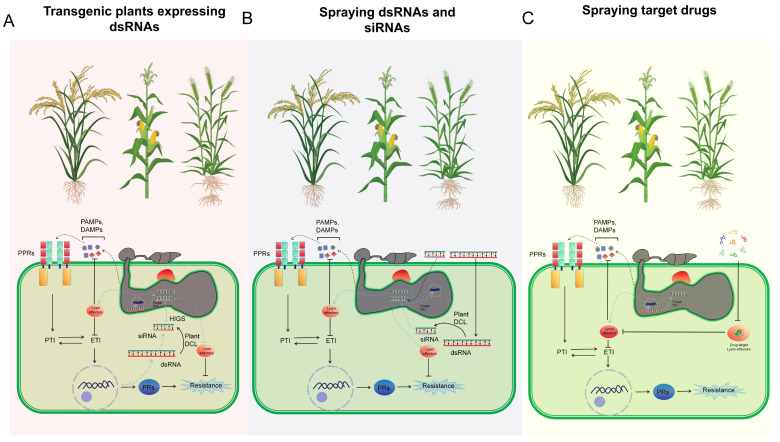
Application of LysM protein research in plant disease control. (**A**) Mechanism of HIGS technology in transgenic plants (e.g., wheat, barley, rice, and maize). Transgenic plants express sequence-specific dsRNAs targeting fungal LysM gene(s). The dsRNAs produced by transgenic plants are cleaved into siRNAs by plant Dicer-like (DCL) proteins. Both uncleaved dsRNAs and siRNAs are transferred into fungal cells when a fungal pathogen infects. The siRNAs in the fungal cells degrade the fungal pathogen mRNAs to counteract pathogen virulence or increase host resistance. (**B**) Mechanism of RNA fungicide targeting LysM domain-containing genes. dsRNAs or siRNAs targeting pathogen gene(s) are sprayed onto plant surfaces and silence pathogen gene(s) through exogenous application. (**C**) Mechanism of chemical fungicide targeting LysM domain proteins. Chemical fungicides targeting pathogen LysM proteins are sprayed onto plant surfaces and inhibit the function of pathogen LysM proteins to reduce virulence.

HIGS is a promising technology for plant disease control, which silences fungal virulence genes by expressing target-specific small RNAs in plants. This strategy has proven effective in multiple pathosystems: for instance, silencing *MoABC1*, *MoMAC1*, and *MoPMK1* in rice enhances resistance to rice blast disease [54,55]; similarly, silencing *CEBiP* or *CERK1* in wheat can restore the virulence of *Z. tritici* even in the absence of a functional *Mg3LysM* gene [43]. Mechanistically, in this strategy, transgenic plants produce double-stranded RNAs (dsRNAs) that are processed into small interfering RNAs (siRNAs) by the plant RNAi machinery; these dsRNAs and siRNAs are then taken up by invading fungi, where they guide the degradation of complementary fungal mRNAs, thereby compromising virulence (Figure 3A). In addition to HIGS, engineering plant PRRs to enhance their chitin-binding affinity or specificity represents another effective strategy for improving plant resistance [56]. Since fungal LysM effectors compete with plant PRRs for chitin binding, modifying plant PRRs to achieve higher chitin-binding affinity than fungal LysM effectors can potentiate CTI [15]. The design principle typically involves mutating key amino acid residues within the LysM domain of CERK1, thereby strengthening its interaction with chitin oligosaccharides and enabling it to outcompete fungal LysM effectors. The feasibility of engineering LysM domain receptors has been demonstrated by constructing chimeric receptors with swapped ectodomains. For instance, replacing the ectodomain of *AtCERK1* with that of the *Lotus japonicus* Nod factor receptors *LjNFR1* or *LjNFR5* enabled the Arabidopsis *cerk1* mutant to perceive rhizobial Nod factors and activate defense responses, including reactive oxygen species (ROS) burst and defense gene expression [57]. This proof-of-concept study suggests that similar approaches could be applied to enhance chitin perception by engineering *CERK1* variants with higher affinity for chitin oligosaccharides, thereby potentially outcompeting fungal LysM effectors. Moreover, structure-guided mutagenesis based on the *AtCERK1*-chitin complex provides a rational basis for designing such high-affinity receptor variants [58].

In contrast to plant defense strategies, many LysM effectors secreted by hemibiotrophic pathogens (HPs) have evolved mechanisms to counteract CTI. These effectors remove chitin oligomers from the host infection site through intermolecular LysM dimerization, or prevent host recognition of chitin via the formation of polymeric precipitates, such as ChElp1 and ChElp2 in *C.higginsianum* [59]. The application of LysM domains for improving plant resistance also includes the use of endolysins to inhibit bacterial growth in transgenic plants, suggesting that chimeric chitinases fused to LysM domains could potentially be exploited to hinder fungal growth in a diverse range of crop plants [60]. Field trials have demonstrated that spray application of dsRNAs targeting the *BcDCL1* and *BcDCL2* genes of *Botrytis cinerea* Pers. significantly suppresses fungal growth and virulence in grapes, reducing lesion size by over 80% [61,62] (Figure 3B). This concept can be extended to develop RNA fungicides specifically targeting LysM domain-encoding genes, representing another promising disease control strategy. Advances in artificial intelligence may enable the design of synthetic peptides or target drugs that mimic chitin and bind to fungal LysM effectors with high affinity, based on the structural features of LysM domains (Figure 3C). This could prevent their interaction with both chitin and plant PRRs, thereby controlling fungal diseases. The main strategies for applying LysM protein research in plant disease control are illustrated in Figure 3. Nevertheless, the application of LysM protein research in plant disease control remains scarcely reported, and further in-depth field studies are urgently required.

## 5. Challenges and Future Perspectives

Despite significant progress in LysM protein research, several challenges remain. First, functional redundancy of LysM effectors in many fungal pathogens makes it difficult to determine their individual contributions to virulence [15]. *Z. tritici* has three LysM effectors with partial functional redundancy, and only the triple mutant shows a significant virulence reduction. *M. oryzae* encodes 12 LysM domain-containing proteins, among which only three (MoSlp1, MoSlp2 and MoCVNH3) have been functionally characterized to date. Although MoSlp2 shows strong similarity to the *C. fulvum* Ecp6 protein, its expression was not detected during plant infection, and no clear phenotype has been observed for Δ*Moslp2* mutants. In addition, the host specificity mechanism of LysM effectors is not fully understood. The lineage-specific LysM effector of *V. dahliae* only contributes to virulence on tomato, but the molecular basis of this host-specificity needs further investigation [17]. Furthermore, the structures of most LysM domain-containing proteins remain unknown, which limits the understanding of their binding specificity and functional mechanisms. Another challenge is that the co-evolutionary relationship between fungal LysM effectors and plant PRRs is complex, and the evolutionary dynamics need further clarification. This includes the emergence of fungal strains carrying mutated LysM effectors that evade LysM-based disease control strategies, as well as the limited understanding of the crosstalk between LysM effectors and other virulence factors.

Future research should focus on the following aspects: (1) Identifying novel LysM effectors and their host targets using multi-omics technologies, such as genomics, transcriptomics, and proteomics, combined with advanced bioinformatics tools like AlphaFold3 [63]. (2) Elucidating the structures of diverse LysM proteins using structural biology techniques, such as X-ray crystallography, and cryo-electron microscopy, to understand their functional mechanisms at the molecular level, especially the host-specific recognition mechanisms. (3) Investigating the host-specificity mechanism of LysM effectors by comparing their functions in different host plants and identifying host factors that mediate this specificity [64]. (4) Exploring the co-evolutionary dynamics between fungal LysM effectors and plant PRRs to clarify the evolutionary arms race between plants and fungi, which can provide insights into the design of durable resistance strategies [65]. (5) Developing more efficient disease control strategies based on LysM proteins, such as optimizing HIGS technology, engineering high-affinity PRRs with broad-spectrum activity, and exploring novel biotechnological approaches, including the development of peptide inhibitors and other protein-targeting strategies. In addition, with the development of AI technologies, predicting the structures and functions of LysM proteins using machine learning will become a new research direction, greatly accelerating the discovery of novel LysM effectors and the elucidation of their functional mechanisms [66]. Furthermore, integrating LysM-based strategies with other disease control methods, such as screening novel fungicides that target LysM domain-containing proteins, will be crucial for achieving sustainable fungal disease management in agriculture.

## 6. Conclusions

LysM domain-containing proteins are key virulence factors in plant pathogenic fungi, and they play diverse roles in fungal pathogenicity by sequestering chitin, inhibiting plant PRR activation, protecting fungal hyphae, and regulating fungal development. Research over the past decade, particularly in recent years, has significantly deepened our understanding of the molecular mechanisms underlying plant-fungal interactions, including the discovery of novel LysM effectors, elucidation of new functional mechanisms, and optimization of LysM-based disease control strategies. Although there are still many challenges, such as functional redundancy of LysM effectors and the emergence of evasive fungal strains, with the continuous development of various research technologies, significant progress will be made in the study of LysM proteins in the future. The application of LysM protein-related research findings in agricultural production has great potential to improve the control efficiency of fungal diseases, reduce the reliance on chemical fungicides, and advance the sustainable development of agricultural systems. With the integration of multi-omics, structural biology, and artificial intelligence technologies, further exploration of LysM protein functions will continue to uncover novel plant-fungal interaction mechanisms and drive the development of innovative, sustainable strategies for fungal disease management in global agriculture.

## Data Availability

No new data were created.
